# Two Polymorphisms Facilitate Differences in Plasticity between Two Chicken Major Histocompatibility Complex Class I Proteins

**DOI:** 10.1371/journal.pone.0089657

**Published:** 2014-02-20

**Authors:** Alistair Bailey, Andy van Hateren, Tim Elliott, Jörn M. Werner

**Affiliations:** 1 Institute for Life Sciences, University of Southampton, Southampton, United Kingdom; 2 Cancer Sciences Unit, Faculty of Medicine, University of Southampton, Southampton, United Kingdom; 3 Centre for Biological Sciences, Faculty of Natural & Environmental Sciences, University of Southampton, Southampton, United Kingdom; The University of Hong Kong, Hong Kong

## Abstract

Major histocompatibility complex class I molecules (MHC I) present peptides to cytotoxic T-cells at the surface of almost all nucleated cells. The function of MHC I molecules is to select high affinity peptides from a large intracellular pool and they are assisted in this process by co-factor molecules, notably tapasin. In contrast to mammals, MHC homozygous chickens express a single MHC I gene locus, termed BF2, which is hypothesised to have co-evolved with the highly polymorphic tapasin within stable haplotypes. The BF2 molecules of the B15 and B19 haplotypes have recently been shown to differ in their interactions with tapasin and in their peptide selection properties. This study investigated whether these observations might be explained by differences in the protein plasticity that is encoded into the MHC I structure by primary sequence polymorphisms. Furthermore, we aimed to demonstrate the utility of a complimentary modelling approach to the understanding of complex experimental data. Combining mechanistic molecular dynamics simulations and the primary sequence based technique of statistical coupling analysis, we show how two of the eight polymorphisms between BF2*15∶01 and BF2*19∶01 facilitate differences in plasticity. We show that BF2*15∶01 is intrinsically more plastic than BF2*19∶01, exploring more conformations in the absence of peptide. We identify a protein sector of contiguous residues connecting the membrane bound α_3_ domain and the heavy chain peptide binding site. This sector contains two of the eight polymorphic residues. One is residue 22 in the peptide binding domain and the other 220 is in the α_3_ domain, a putative tapasin binding site. These observations are in correspondence with the experimentally observed functional differences of these molecules and suggest a mechanism for how modulation of MHC I plasticity by tapasin catalyses peptide selection allosterically.

## Introduction

**Figure 1:**
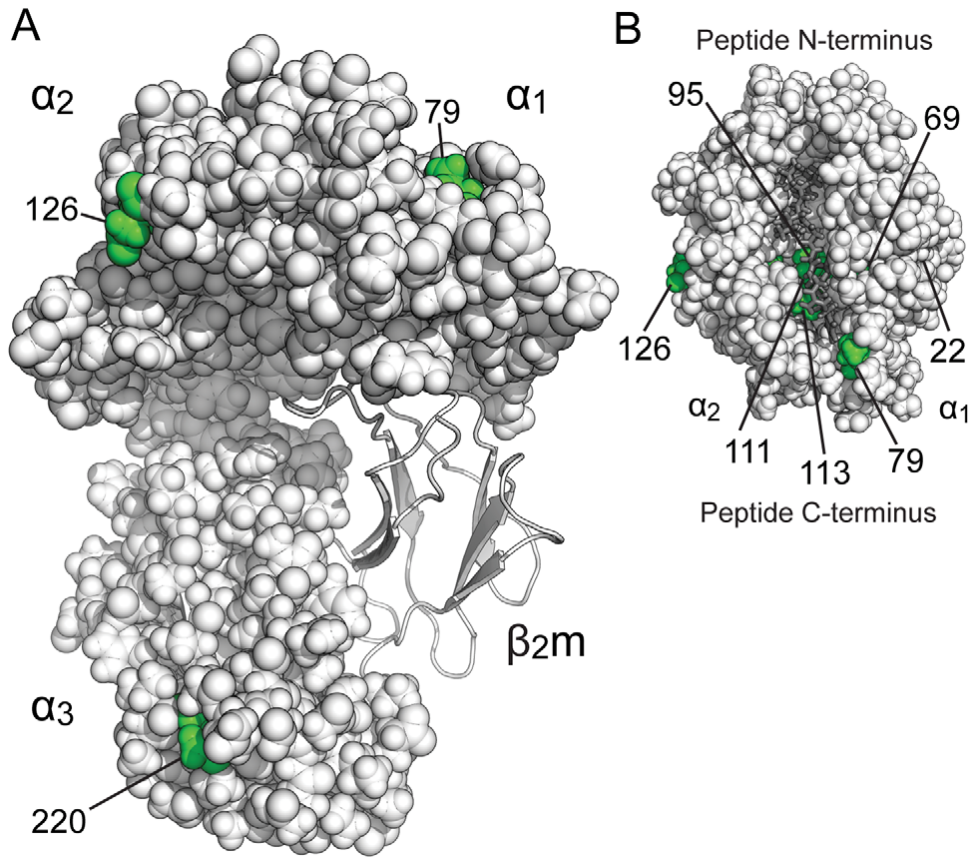
The structure and polymorphisms of chicken MHC Class I alleles BF2*15∶01 and BF2*19∶01. A) The structure of the lumenal domain of a chicken MHC Class I molecule. A space filling representation of the heavy chain is shown, formed of α_1_– α_2_ peptide binding domain and the membrane proximal α_3_ domain, creating a complex with a non-covalently bound β_2_m light chain shown as a ribbon representation. B) The peptide is shown as a stick representation in grey, non-covalently bound into the groove formed between the α_1_ and α_2_ helices. The sites of the polymorphic residues between BF2*15∶01 and BF2*19∶01 indicated in green, with the location of residue 22 indicated in the peptide binding domain below the α_1_ helix.

Major histocompatibility complex class I molecules (MHC I) select peptides for presentation to CD8+ cytotoxic T-cells at the surface of almost all nucleated cells. This MHC I antigen processing and presentation system is a key mechanism in the surveillance and recognition by the immune system of diseased, infected or cancerous cells. Yet understanding how the peptide selection process determines the intensity and specificity of the cytotoxic T-cell response to pathogens remains one of the most important unsolved problems in immunology [1]. Peptides are primarily, but not always, derived from degraded proteins and defective ribosomal products inside the cell and are loaded onto MHC I molecules within the endoplasmic reticulum. As part of this peptide loading complex [2]–[5], MHC I associates with several proteins, most notably the co-factor molecule tapasin, the molecule that most helps MHC I select high affinity peptides [6]–[9]. It is via tapasin that MHC I co-locates with the transporter associated with antigen presentation (TAP) [10] that supplies peptides from the cytosol. MHC class I molecules have a common tertiary structure (Figure 1) consisting of a heavy chain formed of α_1_–α_2_ peptide binding domain and the membrane bound α_3_ domain with a non-covalently bound monomorphic β_2_-microglobulin light chain (β_2_m). Peptides usually of 8–10 amino acids in length bind into the groove formed between the α_1_ and α_2_ helices.

In humans the major histocompatibility complex is a large genomic region spanning approximately 3.5 mega base pairs of DNA nucleotides [11]. It contains genes encoding three classical MHC I alleles that are co-dominantly expressed and are highly polymorphic. The exact reasons for MHC I gene diversity is still unknown, but these genes appear to be at least in part subject to negative frequency dependent, balancing selection processes [12]. That is to say that there is a drive to maintain multiple MHC I alleles, specifically rare alleles, which survive perhaps due to their fitness advantage in presenting pathogen derived peptides. In the human MHC region, the genes for tapasin and TAP are distant from the MHC I genes, have few alleles and exhibit little sequence diversity and have no known functional distinctions. Thus, although in humans TAP favours peptides with hydrophobic C-termini, it has a broad transport specificity [13] and the majority of the specificity for selection of peptide from the available pool is encoded into the MHC I molecule. Likewise tapasin enhances the peptide selection function for all MHC I alleles. The recently characterised TAPBPR molecule may also play a role in the peptide selection process [14].

In contrast to most mammals, chickens have a compact major histocompatibility complex spanning only about 92 kilo base pairs [15]. This contains a single dominantly expressed MHC I gene closely located with the tapasin and TAP genes that are rarely disrupted by recombination events [15], [16]. In chickens the proximity of MHC I, tapasin and TAP genes and the absence of recombination are hypothesised to have led to a diverse set of co-evolving haplotypes with a high degree of allelic polymorphism and sequence diversity of MHC I, tapasin and TAP genes [17], [18]. For certain haplotypes, the peptide specificity of TAP appears to complement the peptide binding motif of the MHC I molecule [19], and tapasin provides complementary enhanced peptide selection functionality, supporting the co-evolution hypothesis.

The chicken haplotypes B19 and B15 express MHC I proteins BF2*19∶01 and BF2*15∶01 respectively which share a similar peptide binding specificity [20], [21], but differ by only eight amino acids in their primary sequences [18]. As shown in Figure 1, seven of these eight polymorphic residues are in the α_1_– α_2_ peptide binding domain, with the eighth polymorphic residue in the membrane bound α_3_ domain. The peptide binding domain residues 79 and 126 and α_3_ domain residue 220 are located on the protein surface, whilst the other polymorphic positions are buried and not immediately accessible. Residues 126 and 220 are located on the surface that is the putative tapasin facing side of MHC I in mammals [22], [23].

We have recently described differences in the abilities of BF2*15∶01 and BF2*19∶01 to select peptide in the presence and absence of both their complementary and mismatched tapasin [24]. This work showed that there are intrinsic differences in the abilities of these MHC I molecules to select high affinity peptides and that the complementary tapasin allele best enhances their selection capabilities *in vivo*. Notably, BF2*15∶01 was less dependent on tapasin for exchange of low affinity peptides for high affinity peptides than BF2*19∶01. Furthermore, this work identified position α_3_ domain residue 220 as relevant to tapasin function and the intrinsic peptide selection properties of these molecules.

Our recent work examining two human HLA–B*44 alleles, that differ by a single amino acid, concluded that it was differences in protein plasticity, the intrinsic ability of the molecule to change shape, that determined the relative dependence on tapasin of these molecules for high affinity peptide selection [unpublished data]. We therefore hypothesised that differences in protein plasticity may also explain the functional differences observed for BF2*15∶01 and BF2*19∶01. We sought to characterise how the polymorphisms between these molecules could alter the plasticity of the MHC I structure in order to rationalise the observed functional differences at the structural level.

We have combined two modelling approaches: mechanistic molecular dynamics simulations [25]–[28] of BF2*15∶01 and BF2*19∶01 and the sequence analysis method of statistical coupling analysis [29], [30]. The aim of this first approach was to use molecular dynamics as a computational microscope [31] to examine whether differences in plasticity arise from the polymorphisms between BF2*15∶01 and BF2*19∶01 as described by their protein dynamics. These dynamics were quantified in terms of sites of local flexibility identified in each MHC I structure, the global motions of each molecule and their relative abilities to explore the conformational space. The aim of the second approach was to identify evolutionarily conserved primary sequence positions in MHC I forming networks of residues, called protein sectors, which are physically connected in the tertiary heavy chain structure. The overarching aim was to identify which heavy chain residues are most likely to encode differences in MHC I plasticity and therefore the biological properties of BF2*15∶01 and BF2*19∶01. Collectively, this created a framework in which to interpret the experimentally observed differences of these MHC I molecules in their intrinsic peptide selection ability and tapasin dependence in terms of protein dynamics, which are reflected in the evolutionary history of their primary sequences.

## Results

Briefly, homology models of BF2*15∶01 and BF2*19∶01 both with and without the 8-mer peptide KRLIGKRY were created using MODELLER [32], [33] based upon the crystal structure of BF2*21 PDB ID: 3BEV [34], and assessed using SWISS-MODEL [35]–[37]. This peptide binds to both molecules with equal affinity [24]. Three independent molecular dynamics simulations of 150 nanoseconds (ns) for each structure were performed using the GROMACS package [25]. Having discarded the first 10ns to remove any effects of the system reaching equilibrium, the final 140ns of each simulation were concatenated to generate a 420ns trajectory for each structure of BF2*15∶01 and BF2*19∶01 in the peptide bound and peptide free state. Residue numbering of these proteins as presented here is for the lumenal domain of the MHC I molecule as found in all PDB structure files i.e. 274 heavy chain residues starting at position 1 after the signal peptide. Further details of the methodology and quality assessment are supplied in materials and methods and supporting information (SI).

### Quantification of the flexibility of BF2*15∶01 and BF2*19∶01 identifies local differences in protein plasticity

To quantify protein plasticity in terms of per residue flexibility during the molecular dynamics simulation we used the conformational angle φ. This is the dihedral angle of internal rotation in the main chain of a protein created by rotation around the N-Cα bond. For each 5 picosecond trajectory snapshot, 84,000 structures over 420ns, the φ angle was measured for each residue in the MHC I complex. The standard deviation of the φ angles therefore quantifies the extent to which each residue varies from their average conformation over the trajectory and thus indicates which regions of the protein are flexible and which are not.

**Figure 2:**
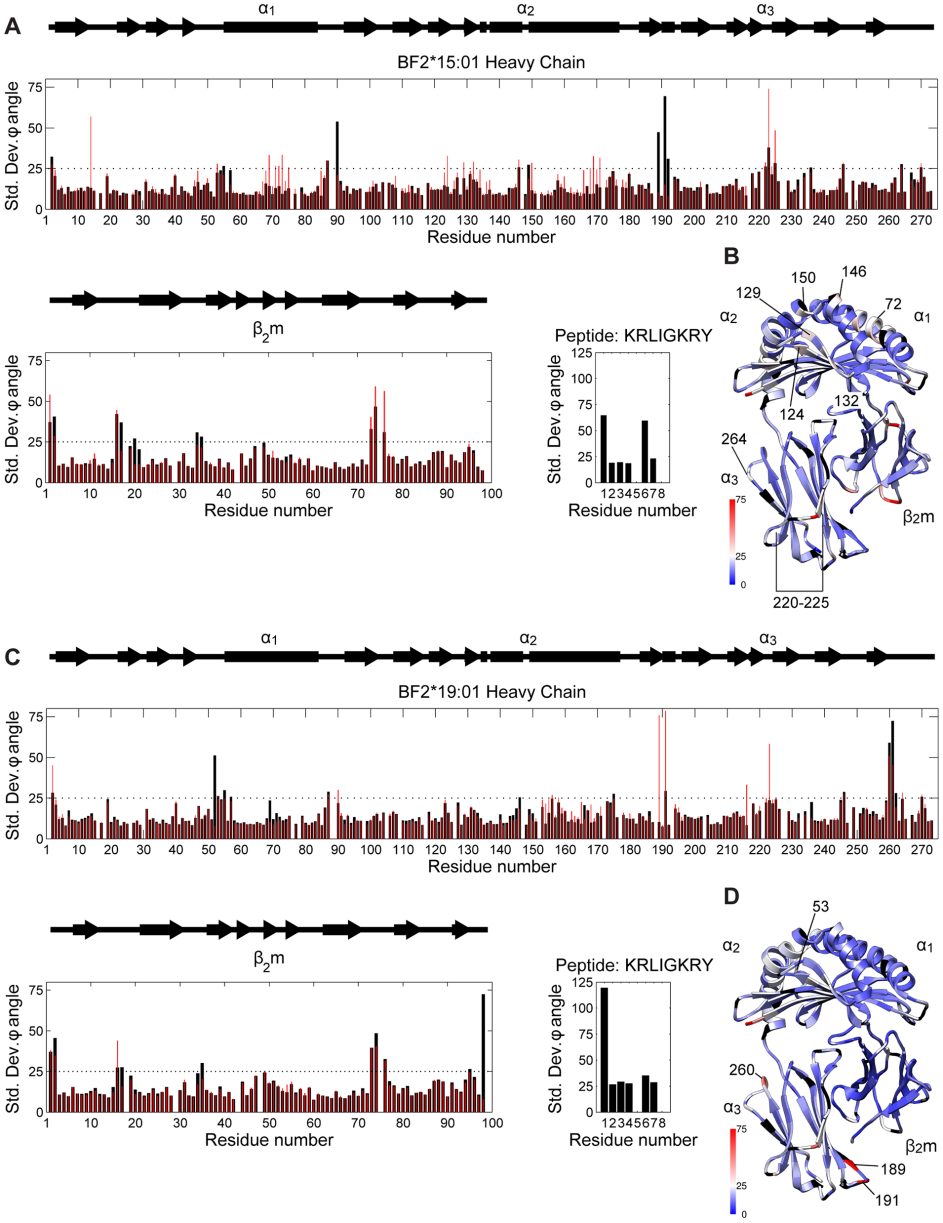
Quantification of the flexibility of MHC I by conformational φ angle standard deviation. A) and C) The standard deviation of the internal angle of rotation φ measuring the rotation around N-Cα bond of each residue of BF2*15∶01 and BF2*19∶01 from 420ns of molecular dynamics simulation in the peptide bound and peptide free states. Peptide bound measurements are shown as black bars and peptide free as red bars. B) and D) Ribbon representations of BF2*15∶01 and BF2*19∶01 with the peptide free simulations φ angle standard deviations mapped as increasing from blue to white to red, with annotations on the BF2*15∶01 heavy chain. Glycine residues are coloured black.

Examination of the peptide bound BF2*15∶01 and BF2*19∶01 molecules φ angle standard deviations revealed that they displayed a similar degree of flexibility (Figure 2A,C). Most residues had φ angle standard deviation below 25°; hence we examined more closely those residues displaying flexibility greater than this threshold. There were 17 and 15 residues in BF2*15∶01 and BF2*19∶01 respectively, with φ angle standard deviations greater than 25°. Unsurprisingly, the unstructured loop regions exhibit the greatest flexibility, such as residues 53 and 90 on loops preceding and following the α_1_ helix respectively. Both molecules have φ angle standard deviations of about 25° in the α_2_ helix around residues 146 and 150 and flanking the peptide N-terminus binding site around residues 168 and 170. Residues around positions 190 and 260 in these hairpin turns in the α_3_ domain indicate that they are highly flexible in both molecules in the peptide bound state.

In contrast, on removal of the peptide there was a marked difference in plasticity between the alleles. We observed an increase in the φ angle standard deviation greater than 25° from 17 to 25 sites on BF2*15∶01 (Figure 2A), but only an increase from 15 to 19 sites on BF2*19∶01 (Figure 2C). In the BF2*15∶01 peptide binding domain, the increased flexibility of residues 132 and 146 in the α_2_ helix flanking the peptide C-terminus binding site suggests these residues might create hinge points about which the helix could rotate (Figure 2B). There was also increased flexibility in α_2_ helix flanking the peptide N-terminus binding site around residues 168 and 170 and in the α_1_ helix around residue 72. In the α_3_ domain there is a decrease in the φ angle standard deviation around the 191 hairpin and a large increase in the loop containing residues 220–225. This is a region of MHC I that is a putative tapasin binding site (Figure 2B).

For BF2*19∶01, upon removal of the peptide, the largest increases in flexibility were observed in the residues around 191 hairpin in the α_3_ domain, in contrast to BF2*15∶01. In common with BF2*15∶01 there was an increase in flexibility around residue 222 in the putative tapasin binding loop in the α_3_ domain (Figure 2C,D). These changes coincided with the flexibility of the peptide binding domain of BF2*19∶01 remaining broadly as in the peptide bound state, whereas BF2*15∶01 became more plastic on removal of the peptide.

Overall, this measure of local flexibility suggested that the BF2*15∶01 heavy chain has a more intrinsically plastic structure than the BF2*19∶01 heavy chain, and so next we looked for further evidence of this difference in the global dynamics of these proteins.

### Identification of the global motions of BF2*15∶01 and BF2*19∶01 and conformational exploration by Principal Component Analysis

To understand how local flexibility impacts upon plasticity in terms of the global dynamics of these MHC I molecules we used Principal Component Analysis (PCA). PCA aims to identify the modes of motion corresponding to the directions along which the covariance of backbone atomic motions during the simulation are maximised. This is to say that in contrast to examining residues individually, as with the conformational angle analysis, we identify the collective motions of the atoms in MHC I and rank them according to their contribution to the overall motion i.e. how principal each component is. The underlying assumptions are that the dynamics of MHC I are best expressed in terms of a few modes containing large variances and that these are relevant to function [38], [39]. Here PCA provides us with three pieces of information about the dynamics of BF2*15∶01 and BF2*19∶01: 1) PCA identifies and quantifies which collective atomic motions most contribute to the overall motion of the molecule during simulation. 2) We can project the dominant collective motions onto the MHC I structure to observe their quality and compare them between molecules. 3) Having identified the dominant motions, we can also examine how many different conformations are explored by these collective motions, and how frequently each conformation occurs. Further details of PCA analysis are provided in the materials and methods. It is important to note that one would not expect the φ and PCA analyses to be directly correlated on a residue by residue basis. Indeed, the rationale for using the different analyses presented is to try and build a detailed picture of the dynamics from different perspectives. For example, a residue that shows great local flexibility may not necessarily undergo large amplitude motions; rather it may be a residue whose flexibility facilitates other residues to undergo large amplitude motions. Lack of direct correlation between local flexibility and the amplitude of motion for a given residue is therefore not unexpected.

**Figure 3:**
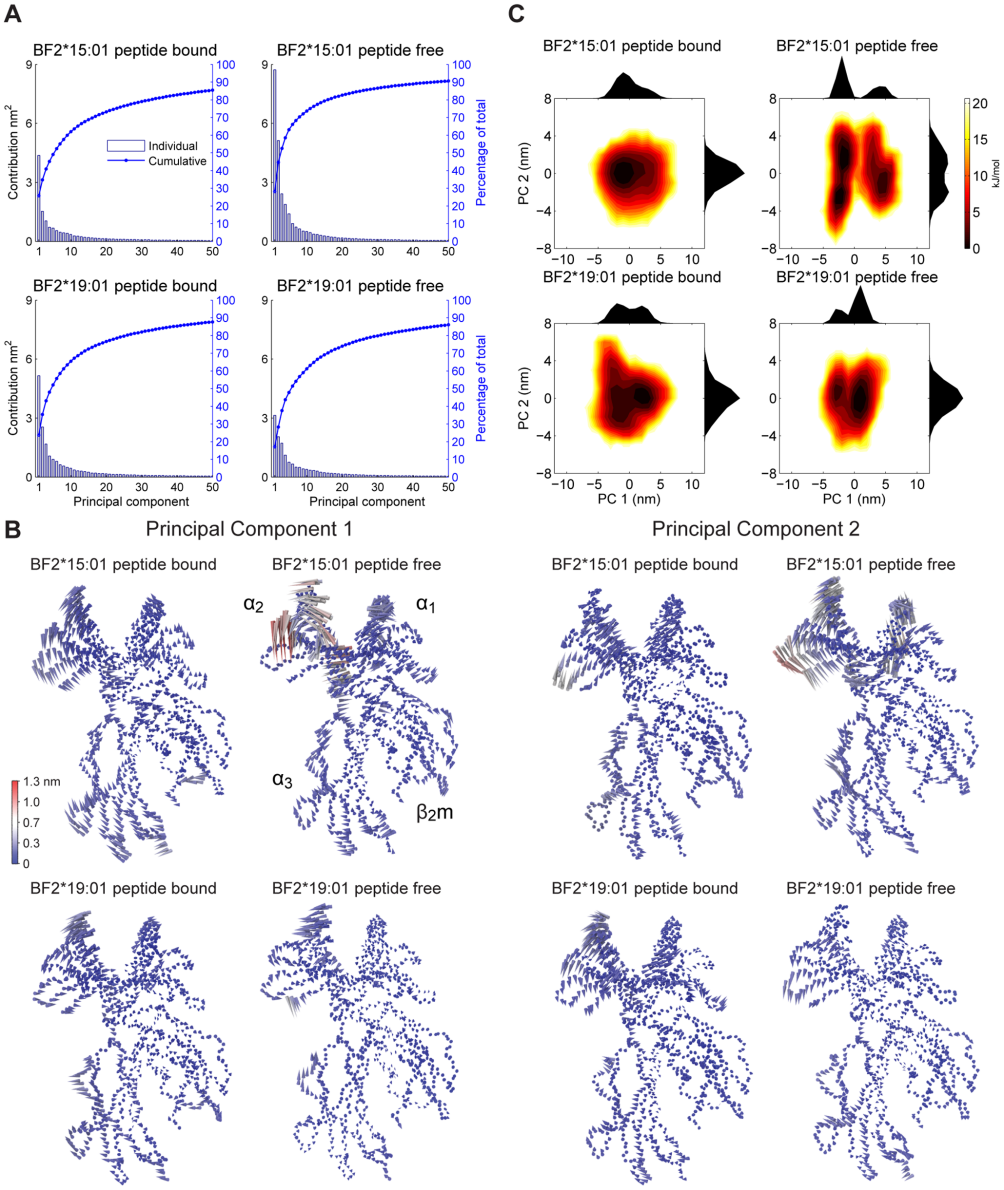
The global dynamics of MHC I identified by Principal Component Analysis. For each 420ns molecular dynamics simulation of BF2*15∶01 and BF2*19∶01 PCA was performed using a common peptide free backbone structure. A) Contributions of the first 50 PCs to the total variance of the backbone atomic motions. B) Porcupine plots indicate the magnitude and direction of motion for each backbone atom along PC1 and 2 in both the peptide bound and peptide free states. The magnitude between extremes is indicated by the colour bar. C) Gibbs free energy landscapes are generated from the principal coordinates of PC1 and PC2 and transformed by treatment as a Boltzmann ensemble. Individual probability densities for PC1 and PC2 are plotted on the outside adjacent axes.

Therefore, we first calculated the variance contributed by each individual principal component (PC), and the percentage of total variance accounted for by the PCs cumulatively (Figure 3A). It is clear that the first 50 PCs are sufficient to describe almost all of the backbone atomic motions in all simulations. For BF2*15∶01 the first two principal components account for about 35% of the total variance in the peptide bound state with nearly 30% contained within the dominant PC1 mode. This increases to about 45% in the peptide free state, corresponding with nearly a doubling of the actual backbone variance of these modes. For BF2*19∶01 the first two principal components also account for about 35% of the total variance in the peptide bound state, with about 25% contained within the dominant PC1 mode. The contribution of the first two PCs to the total variance falls to below 30% on the removal of the peptide, with nearly a halving of the variance contributed by PC1. This suggests that both molecules have a similar plasticity as quantified by PCA in the native peptide bound state, but display contrasting degrees of plasticity in the non-native peptide free state in correspondence with the overall differences observed between the molecules in the φ angle standard deviation analysis.

To then examine these similarities and differences, qualitatively as well as quantitatively, the top two principal components were visualised as porcupine plots showing the direction and magnitude of the motion of each backbone atom along PC1 and PC2 (Figure 3B, Movies S1). In the peptide bound state, the magnitudes of the atomic fluctuations are similar for both BF2*15∶01 and BF2*19∶01 along both the modes PC1 and PC2. However, qualitatively they are different. The BF2*19∶01 heavy chain domains have a twisting mode for PC1 whilst BF2*15∶01 displays a swinging motion between heavy chain domains. Both molecules show twisting dynamic between domains for the heavy chain PC2 mode.

On removal of the peptide both molecules display the same quality of heavy chain motions for PC1 and PC2, but the amplitudes of the motions are greater for BF2*15∶01 than BF2*19∶01. Dominant mode PC1 describes an opening and closing of the helices flanking the peptide binding groove corresponding with a twisting between heavy chain domains centred about the domain-domain linker region. This suggests that the motions of the heavy chain domains are dynamically coupled [40]. In other words large domain-domain motions appear to correspond with large conformational changes in the peptide binding domain and vice versa. The peptide free PC2 mode describes a combined rocking and twisting motion between peptide binding groove and heavy chain domains. For BF2*15∶01 the PC1 motion is pronounced with the greatest amplitudes occurring in the α_2_ helix between residues 134 and 150 that flank the peptide C-terminus binding site. In contrast, BF2*19∶01 PC1 shows a reduction in the amplitude of domain-domain motions as compared with the peptide bound state. For PC2 BF2*15∶01 again has much greater amplitudes than BF2*19∶01 with the largest motions in the peptide binding domain helices.

To examine the extent to which these molecules are actually able to explore the different conformations indicated in the porcupine plots, the simulation trajectories were treated as Boltzmann ensembles and plotted as Gibbs free energy landscapes (Figure 3C). This was done by calculating a transformation of the joint probability distribution of the coordinates of the top two dominant modes. This information about how BF2*15∶01 and BF2*19∶01 explore their conformational landscapes further indicates any differences in plasticity by indicating how likely they are to populate different conformations. In the peptide bound state both molecules inhabit a single energy minimum indicating the stability of the peptide bound state. Thus although we observe the possibility of various conformations in the porcupine plots (Figure 3B), these landscapes suggest that in fact peptide bound MHC I mostly inhabits a single conformation and infrequently visits other states.

On the removal of peptide from BF2*15∶01, the molecule explores a larger region of the energy landscape and populates three local energy minima. The probability distribution broadens along PC2 and forms a two peaked distribution along PC1 with a dominant and sub-dominant peak separated by a large energy barrier. In one half of the landscape is a minima corresponding with the sub-dominant PC1 probability distribution peak. On the other half of the landscape, there are two distinct energy minima corresponding with the dominant PC1 probability distribution peak, separated by a small energy barrier along the PC2 axis. In contrast to BF2*15∶01, removal of the peptide from BF2*19∶01 leads to the appearance of two closely located energy minima, but with an increased energy barrier between these states and less of the landscape explored overall.

In summary, PCA indicates that in absence of peptide BF2*15∶01 is more able to explore different conformations, consistent with the suggestion from the φ angle analysis that the BF2*15∶01 molecule is more plastic than BF2*19∶01 (Figure 3C). When peptide is present both molecules display similar dynamics and plasticity, also consistent with the φ angle analysis. Furthermore the difference in the plasticity between BF2*15∶01 and BF2*19∶01 in the absence of peptide manifests itself as a greater intrinsic ability of the BF2*15∶01 molecule to explore peptide binding groove conformations, and that these motions are coupled to the motions of the α_3_ domain (Figure 3B).

### Statistical coupling analysis identifies a protein sector spanning the peptide binding domain and the α_3_ domain in chicken MHC class I

**Figure 4:**
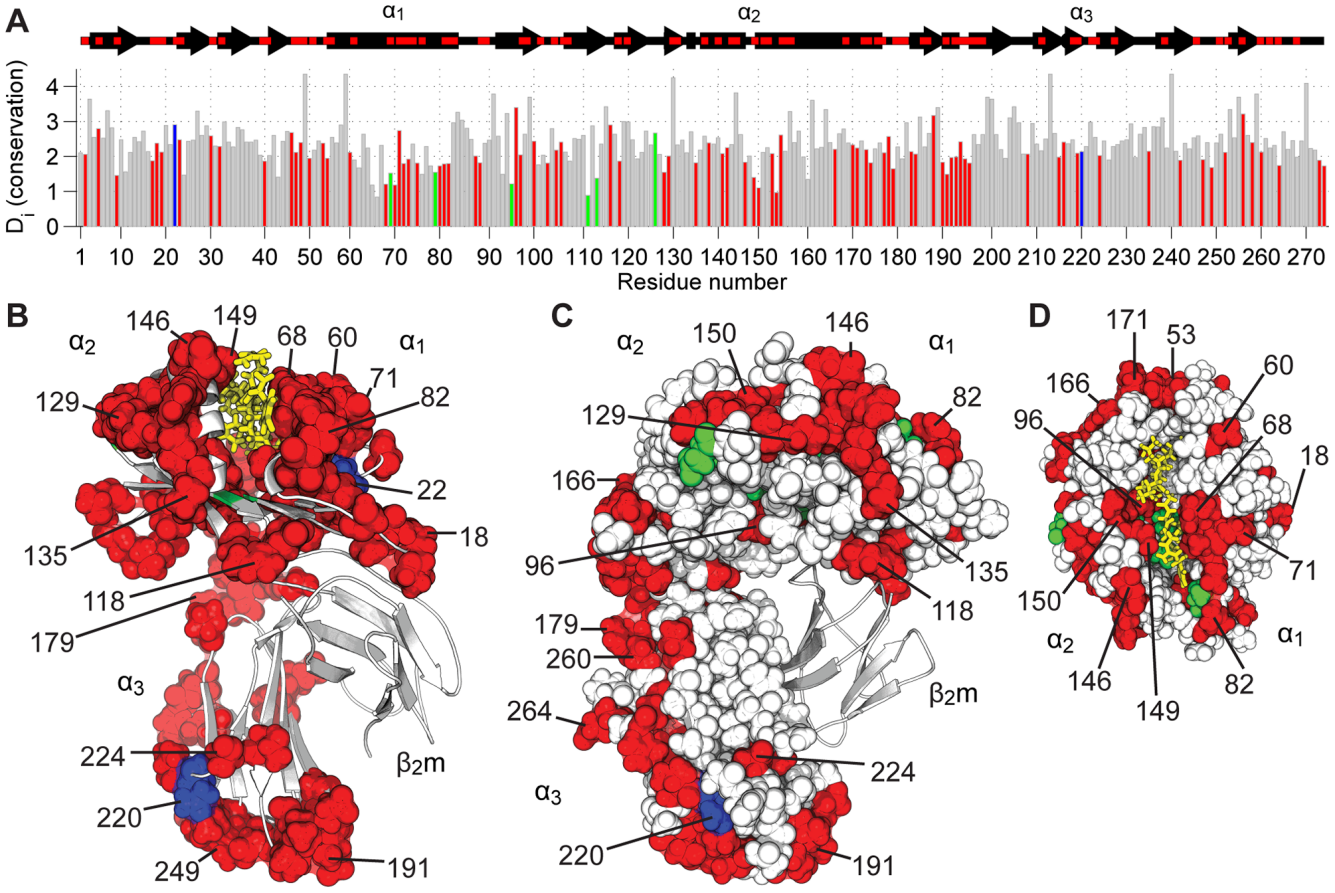
Identification of a protein sector in chicken MHC I. Statistical coupling analysis (SCA) was carried out on a multiple sequence alignment (MSA) of 141 sequences obtained from a similarity search querying the BF2*15∶01 heavy chain as described in [30]. A) The degree of conservation of each heavy chain residue i in the MSA is computed as the Kullback-Leibler relative entropy D_i_. Bigger bars indicate greater conservation. The 85 protein sector residues are in red, 6 polymorphic residues between BF2*15∶01 and BF2*19∶01 are in green and the 2 residues that are both polymorphic and part of the protein sector are in blue. All other residues are in grey. B) Protein sector residues are mapped as spheres onto a ribbon representation of the BF2*15∶01 structure. Colours as (A), with the peptide as yellow sticks. C) and D) Space filling representations of the MHC I heavy chain, coloured as (B). The contiguous network of residues forming a protein sector comprises of 31% of heavy chain residues.

Finally we sought to identify positions in the primary amino acid sequences of BF2*15∶01 and BF2*19∶01 that could form networks of residues through which the observed dynamics and differences in plasticity act. To this end we used the previously described technique of statistical coupling analysis (SCA) [29], [30], [41]–[43] to identify non-random correlations between sequence positions of a multiple sequence alignment (MSA), generated using BF2*15∶01 heavy chain as the query sequence. To briefly summarise, using a MSA of 141 sequences (MSA S1), the positional conservation of each heavy chain residue was calculated (Figure 4A). This conservational weighting was then used to calculate a matrix of correlations between all pairs of MSA positions to quantify the evolutionary history of each pair of sequence positions in the alignment. Eigenvalue decomposition of this positional correlation matrix identified the top six statistically significant eigenmodes describing weighted groups of positions (Figure S1). Using Independent Component Analysis (ICA) as previously described [30], we transformed these eigenmodes and projected the heavy chain positions along three maximally independent axes (Figure S2). One of these directions, IC2, identified a group of heavy chain residues that was used to define a single MHC I heavy chain protein sector (Figure S2). Further details are provided in the materials and methods and in Refs [29], [30], [41]–[43].

Here SCA identified a protein sector that creates a contiguous network of 85 residues, constituting approximately 31% of heavy chain residues, connecting the peptide binding domain and the α_3_ domain of the chicken MHC I heavy chain (Figure 4, Sector S1). The sector connects residues along the α_1_ helix (Figure 4A–D) and passes across the peptide binding groove via residue 96 at the interaction site of β_2_m (Figure 4C) to connect with the α_2_ helix. Connection to the α_3_ domain is through the domain linker residues 177, 178 and 179 (Figure 4A–C) and the sector continues almost to the region of the heavy chain that would ordinarily connect to the transmembrane domain (Figure 4A–C). Importantly, this protein sector also includes many of the residues identified as sites of local flexibility in φ angle standard deviation analysis, such as 71,129 and 150 in the peptide binding domain and α_3_ domain residues 191, 224 and 264 (Figure 1B and 4). This sector is also consistent with the notion that MHC I heavy chain domains are dynamically coupled as suggested from the PCA analysis.

Of the eight polymorphic residues that differ between BF2*15∶01 and BF2*19∶01, coloured green in Figure 4, two are identified as sector residues, coloured blue in Figure 4. These polymorphic residues are position 22 in the peptide binding groove just below the α_1_ helix and position 220 in the α_3_ domain (Figure 4A–C). We have recently shown that when amino acid position 220 is swapped between BF2*15∶01 and BF2*19∶01, their ability to exchange low affinity peptides for high affinity peptide *in vitro* is altered [24]. This observed allosteric effect on peptide editing may be explained at the molecular level by this sector spanning the peptide binding domain and the the α_3_ domain. Interestingly, whilst mutation of position 220 had a detrimental effect on this measure of intrinsic peptide selection ability for both alleles, mutation of non-sector position 126 did not. Furthermore, position 220 is known to reside in, or near, a putative tapasin interaction site [44], [45] and the polymorphism at position 220 influenced the extent to which each allele benefited from presence of tapasin when these assays were repeated. Alongside the dynamics simulation data, these observations suggest that by interacting with position 220 on MHC I, tapasin exerts an allosteric influence on the peptide binding domain via the protein sector we have identified.

These observations give the first indication of how we might connect the protein dynamics to the observed functional differences of BF2*15∶01 and BF2*19∶01. Here we have identified a sector in MHC I of 85 sequence positions that have been evolutionarily conserved and containing just two of the polymorphic residues. These polymorphisms at positions 22 and 220 correspond with the differences in their intrinsic plasticity indicated by the changes in dynamic coupling between BF2*15∶01 and BF2*19∶01 seen during the peptide free molecular dynamics simulations. This sector therefore indicates which residues through which dynamic coupling may occur in MHC I and suggests a link between polymorphisms and changes in protein dynamics.

## Discussion

The aim of the study presented here was to examine the protein dynamics of two chicken MHC I molecules in the light of the co-evolving haplotype hypothesis [19] and experimentally observed differences in the intrinsic peptide selection abilities of these alleles [24]. The co-evolving haplotype hypothesis proposes that in chickens, unlike in mammals, the genes for MHC I, tapasin and TAP have evolved together with optimal function resulting from alleles of these proteins encoded from the same haplotype that share complementary functions. Recently, this hypothesis was tested experimentally using the same MHC I molecules presented in this study, BF2*15∶01 and BF2*19∶01 [24] which proposed a mechanistic basis for the co-evolution of chicken tapasin and MHC I molecules. These experiments demonstrated that the mismatching of MHC I and tapasin molecules from these haplotypes impaired the maturation of MHC I *in vivo*. Secondly, as described in the previous section, differences in the intrinsic peptide selection properties of BF2*15∶01 and BF2*19∶01 in the absence of tapasin *in vitro* were also observed. Importantly, position 220 in the α_3_ domain was shown to influence these intrinsic properties as well as tapasin function.

Both BF2*15∶01 and BF2*19∶01 have a similar specificity for the peptides that they can bind [20], [21], so the hypothesis we examined here was whether the differences observed *in vitro* and *in vivo* could be a consequence of differences in protein dynamics arising from the polymorphisms. Furthermore we wanted to test the utility of computational models in deepening our understanding of complex biological data and provide a rational framework for future investigations.

The work presented here relies entirely upon models, so the initial limitation we must address is:

### 1. The reliability and limitations of homology models and molecular dynamics simulations

Our analysis of the plasticity of BF2*15∶01 and BF2*19∶01 made use of both homology models of these molecules and molecular dynamics simulations. The limitations on these modelling methods are firstly the quality of the models generated; the timescales available and the approximations made in the treatment of the molecules in the molecular dynamics simulations.

Assessment of the quality of the homology models using SWISS-MODEL [35] indicates they exhibit a similar degree of quality to the X-ray structure on which they are based (Figure S3). Similarly the molecular dynamics simulations have been assessed for stability of the trajectories using time block analysis of the root mean square fluctuations of the average atom positions (Figure S4). The force field used here [28], [46] demonstrated good agreement between simulated folding events and experimentally observed structures in recent investigations into protein folding. This is important as a further complication is the removal of the peptide which creates non-physical structures that cannot be directly compared against experimental observation. So whilst these simulations may not be a true representation of reality, they are consistent with the behaviour of proteins where more direct comparison has been possible. Lastly, here the simulations explore ∼0.5 μs timescale which is below the >1 μs timescale on which we might expect large dynamic events to occur [47], however they do provide an indication of what protein dynamics are possible.

### 2. BF2*15∶01 and BF2*19∶01 exhibit differences in protein plasticity

Analysis of the local flexibility of BF2*15∶01 and BF2*19∶01 using φ angle standard deviations and the global dynamics using PCA indicates that these molecules do differ in their intrinsic plasticity. BF2*15∶01 appears to be an intrinsically more plastic protein than BF2*19∶01 in the absence of peptide. On removal of the peptide we observe a greater increase in the number of local sites of flexibility for BF2*15∶01 than BF2*19∶01 (Figure 2). This proposal is supported by the PCA analysis of the global dynamics. Here we observe that the molecules display similar dynamics in the peptide bound state both quantitatively and qualitatively and mostly occupied a single conformational state. This is consistent with the observation of a stable conformation for MHC I seen in crystallographic structures. On removal of the peptide, BF2*15∶01 explores multiple conformational minima (Figure 3C) corresponding with an increase in the ability to explore a range of peptide binding groove conformations correlated with domain-domain motions (Figure 3B). Conversely, peptide free BF2*19∶01 displays a decrease in the global motions described by the top two modes and explores less of the energy landscape than peptide free BF2*15∶01.

As previously described [38] there are several assumptions and limitations to PCA analysis: One is the assumption that the dynamics most important for protein function are described by the first few principal components which contain the largest variances. Another is that this is a linear analysis and therefore it neglects motions the may be spread across several components. However there are many examples in the literature supporting the notion that the top principal modes do contain functional motions, such as for T4 lysozyme [48].

Whilst the peptide free states represent non-physical structures, the notion of conformational intermediates for MHC I has been hypothesized on the basis of several pieces of circumstantial experimental evidence [49]–[51]. These simulations do not refute that proposal and further inform how they might arise via the plasticity encoded in the primary sequences of these alleles.

### 3. Modulation of protein plasticity by MHC I polymorphisms

Analysis of the MHC I heavy chain using SCA revealed an allosteric protein sector, connecting residues in both helices of the peptide binding domain, passing across the base of the ligand binding site at a site of interaction with β_2_m, and down through the domain linker into the α_3_ domain. Importantly, the sector contains only two of the eight polymorphisms that exist between BF2*15∶01 and BF2*19∶01. One sector polymorphism is in the peptide binding domain beneath the α_1_ helix at position 22 and the other in the α_3_ domain at position 220 (Figure 4). These observations suggest a role for these residues in modulating differences in protein plasticity when compared to the molecular dynamics simulations. The molecular dynamics simulations indicate that these polymorphisms are capable of modulating the amplitude of domain coupled backbone motions (Figure 2B), but do not inform us as to their relative importance. The protein sector identifies a possible network through which the dynamics may act and two residues that may modulate the differences between BF2*15∶01 and BF2*19∶01. What is most striking is that this analysis identifies position 220 as a sector residue. This was identified in the *in vitro* experiments as influencing the ability of tapasin to enhance peptide dissociation, a critical component of MHC I peptide selection. Exchange of position 220 between BF2*15∶01 and BF2*19∶01 influenced the magnitude of tapasin function, suggesting a possible mechanism for how tapasin might modulate the peptide binding groove conformation via the α_3_ domain and catalyse the selection of high affinity peptides by MHC I. The significance of position 22 in the peptide binding domain is not obvious, but intriguing. Our speculation is that a more intrinsically plastic molecule, such as we propose for BF2*15∶01, is better able to sample the conformational space and more quickly select a high affinity peptide than a less plastic molecule and is therefore less reliant on tapasin to access the relevant conformations, and vice versa. Alternative approaches to sector identification using conformational angles from molecular dynamics trajectories have been demonstrated [52], [53]. In this approach, the measurement of the mutual information between the side chain conformations, rotameric states, of each residue in the molecular dynamics trajectory looks for evidence of local or distant coupling between residues. The mutual information for two residues measures the extent to which the rotameric state of one residue depends upon the rotameric state of the other. Thus, as has been shown for β-lactamase [54], a mutual information matrix can be constructed for all the residues in a protein to indicate dynamically coupled residues that may form a sector. Robust measurement of mutual information of rotameric states using the approach described in [54] requires a large ensemble of structures far greater than what is presented here, but this would be an important line of investigation for future work in terms of cross-validation of the SCA.

### 4. Evidence that protein plasticity and protein dynamics are evolutionarily conserved features of MHC I

Our identification of a protein sector indicates how a subset of conserved heavy chain residues connect to form a contiguous network throughout the MHC I heavy chain. Alongside the molecular dynamics data, this suggests the possibility that protein dynamics are a conserved feature of MHC I molecules. SCA has previously been used to identify an allosteric sector in Hsp70 proteins [30]. This showed how two functional protein domains could be coupled, and thus conserved, through a network of connecting residues. The implication of this finding was that there exists a dynamic mechanism in Hsp70 molecules operating through this sector. Our work supports the notion that protein dynamics are conserved features of proteins that are encoded into the primary sequence and which underpin biological function. This is most apparent from the observation that BF2*15∶01 and BF2*19∶01 have similar protein dynamics in the peptide bound state, that is after they have achieved their function, but different dynamics in the peptide free state, prior to peptide selection.

These observations, alongside our recent work with human HLA-B44 alleles [unpublished data], have led us to believe that protein plasticity is an important determinant of MHC class I function. Moreover, this work focuses and complements experimental investigations into the mechanism of peptide selection by MHC I by reducing the target for future investigation from 274 heavy chain residues to 85. Our expectation is that understanding the mechanism by which protein plasticity manifests intrinsically and is modulated by co-factor molecules such as tapasin will be a key part of understanding the peptide selection process determining the immune response in other species, including humans. We foresee establishing techniques such as molecular dynamics and SCA as fully integrated tools in the investigative process as a means of accelerating these developments.

## Materials and Methods

### Homology modelling and molecular dynamics simulations

The starting conformation of BF2*15∶01 and BF2*19∶01 were derived from the experimentally determined structure from the RSCB Protein Databank of BF2*B21, PDB id: 3BEV [34]. These homology models were created used MODELLER [32], [33]. Quality assurance of these models was performed with SWISS-MODEL incorporating PROCHECK [35]–[37].

The GROMACS version 4.5.3 [25] molecular dynamics package was used for the all atom simulations. The simulations used the Amber99SB-ILDN [28] force field and TIP3P [55] explicit water molecules using the Simple Point Charge water system [56], and Sodium counter ions were added to neutralise the charge of the system. The protein structures were placed in rhombic dodecahedron shaped box centred at 1.5 nm from the edge with periodic boundary conditions. Covalent bond lengths were constrained using the P-LINCS algorithm [57] and the water angles were constrained using the SETTLE algorithm [58] allowing an integration time step of 2 fs to be used. Nosé-Hoover temperature coupling [59], [60] and Parinello-Rhaman pressure coupling [61], [62] used a time constant of 0.5 ps with reference baths of 300 Kelvin and 1 bar respectively to maintain the average thermodynamic properties of the protein and solvent comprising the system. Electrostatic interactions use a cut-off of 1 nm with the interactions beyond this cut-off treated using the particle mesh Ewald method [63]. Van der Waals forces used a cut-off of 1 nm. The neighbour list is updated every five steps. Each system initially underwent an energy minimization over 1000 steps of 2 fs to relax the structure and remove the forces from the systems that were introduced by the protonation of the molecule and addition of solvent. This was followed by a 5 ns equilibration of the water surround the protein with the protein atoms restrained using a randomly generated initial starting velocity. Full production runs were performed with the position restraints released. To analyse conformational dynamics, concatenated trajectories of 420 ns were created from three independent repeats of 150 ns, with the first 10 ns of each simulation discarded. Quality assurance and post processing, including PCA, was performed using a combination of the suite of utilities provided with GROMACS. Additional post processing tasks were performed using MATLAB™ and bespoke UNIX awk scripts. Visualisation of the protein structures and molecular dynamics trajectories was performed using the VMD [64] and USCF Chimera [65] packages.

### Principal Component Analysis

Principal component analysis is performed as follows using the GROMACS g_covar and the g_anaeig utilities:

A mass weighted variance-covariance matrix is built using the backbone atoms. This is a symmetric 3N ×3N matrix comprising of the fluctuation of the atom positions with coordinates x as a function of the trajectory t such that:
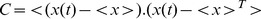
(1)where <> indicates the conformational ensemble average. This matrix C therefore contains as elements, for each atom pair, the difference between the mean product of their atomic positions and the product of their mean atom positions i.e. the difference between their average position as a pair and the product of their individual average positions. Atom pairs moving together in the same direction give rise to positive covariances and pairs moving in the opposite direction give rise to negative covariances. Non-correlated atoms give near zero covariances. The variance for each atom is contained on the main diagonal.

With reference to the covariance matrix C generated by equation 1, Eigen decomposition of matrix C is performed using the eigenvector matrix P, its inverse P^−1^ and the diagonal matrix D which has the corresponding eigenvalues on the diagonal, such that:
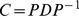
(2)


The eigenvalues along the diagonal in D represent the mean square fluctuations for each eigenvector in C (the columns of P) and therefore indicate how much each eigenvector contributes to the total fluctuation. The eigenvectors are sorted according to size of the eigenvalues. Projection of the data onto the first eigenvector transforms the data into a new coordinate system with the greatest variance residing on this first coordinate. This coordinate is called the first principal component and the first eigenvector is also known as the first principal mode. This projection can be done for each principal mode µ_i_ to yield the principal components as a function of the trajectory p_i_(t) as follows:
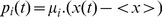
(3)


The variance of principal component <p^2^_i_> is equal to its corresponding eigenvalue in D. The projections can then be transformed back into Cartesian coordinates, x' _i_(t) for visualisation by rearranging equation 3 such that a linear equation describes the coordinates x as a function of the trajectory in terms of the principal coordinates and the average ensemble coordinates <x>:
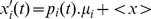
(4)


### Statistical Coupling Analysis

Statistical coupling analysis (SCA) was carried out on a multiple sequence alignment (MSA) of 141 sequences generated using BF2*15∶01 heavy chain as the query sequence for a PSI-BLAST [66] search of the non-redundant UniProtKB/SwissProt sequences database (July 2013), using the BLOSUM62 scoring matrix and an expectation threshold of 0.0001. Automatic alignment of the search results was performed with Clustal Omega [67], [68] followed by manual alignment using SeaView [69].

SCA was performed using a process described in [30], [41] and implemented in the SCA 5.0 toolbox for MATLAB^TM^ available from the Ranganathan laboratory website: http://systems.swmed.edu/rr_lab/sca.html. The sector was defined by empirical fitting of the Students t-distribution to a histogram of the positional weights along IC2 with a cumulative probability density cut-off in the tail of the distribution of 85%. No mechanistic basis is implied by the use of this distribution and the choice of cut-off is that which produces a sector comprising a contiguous network of residues in the tertiary structure. These top 15% of positions in the IC2 distribution represent about 31% of the heavy chain residues, consistent with previous definitions of protein sectors [41].

## Supporting Information

Figure S1 **Histograms of the eigenvalues of the SCA positional correlation matrix.** Histograms of the eigenvalues from decomposition of the positional correlation matrix for the BF2 MHC I heavy chain multiple sequence alignment in blue. The top six eigenmodes are indicated with arrows. Eigenvalues generated from decomposition of 100 randomized alignments are shown in red.
(TIF)

Figure S2 **Identification of the protein sector by Independent Component Analysis.** A plot of the top three independent components generated by transformation of the top six eigenmodes of the SCA matrix using Independent Component Analysis as previously described in [29], [41] to test for the existence of quasi-independent sectors. The identified protein sector is indicated along IC2 in red. The polymorphisms between BF2*15∶01 and BF2*19∶01 are shown in green. Two pusedo-sectors identified by the ICA are indicated in cyan and magenta. These putative sectors were discarded as they are not contiguous in the tertiary structure and most residues are close to zero.
(TIF)

Figure S3 Ramachandran plots comparing the homology models of BF2*15∶01 and BF2*19∶01 to the template structure of BF2*21. Ramachandran plots indicating the conformational φ and ψ angles to assess the quality of the homology models of A) BF2*15∶01 and B) BF2*19∶01 in comparison to the crystallographic template structure of C) BF2*21 were generated using SWISS-MODEL incorporating PROCHECK [34]–[37].
(TIF)

Figure S4 Time block assessment of the stability of the molecular dynamics simulations of BF2*15∶01 and BF2*19∶01. Each plot shows the Root Mean Square Fluctuation (RMSF) of the atoms from their average position during each 10 nanosecond time block of each molecular dynamics simulation trajectory as an indication of the overall stability of each simulation and between simulations.
(TIF)

MSA S1 **The multiple sequence alignment used for the SCA in fasta format.**
(FA) Click here for additional data file.

Sector S1 **The protein sector identified by SCA for BF2 heavy chain using PDB residue numbering.**
(DOC)

Movies S1 Animations of molecular dynamics simulations of BF2*15∶01 and BF2*19∶01 projected onto the first two principal components. The peptide is removed so that a common structure is used for the projections. The magnitude of the motions for BF2*15∶01 peptide free PC1 has created the appearance of a broken molecule. This is an artefact of the rendering.
(PDF)
