# Supplementary figures and images for: Two Polymorphisms Facilitate Differences in Plasticity between Two Chicken Major Histocompatibility Complex Class I Proteins
Source: PLoS One. 2014 Feb 20;9(2):e89657. doi: 10.1371/journal.pone.0089657 (PMC3930747; doi:10.1371/journal.pone.0089657)

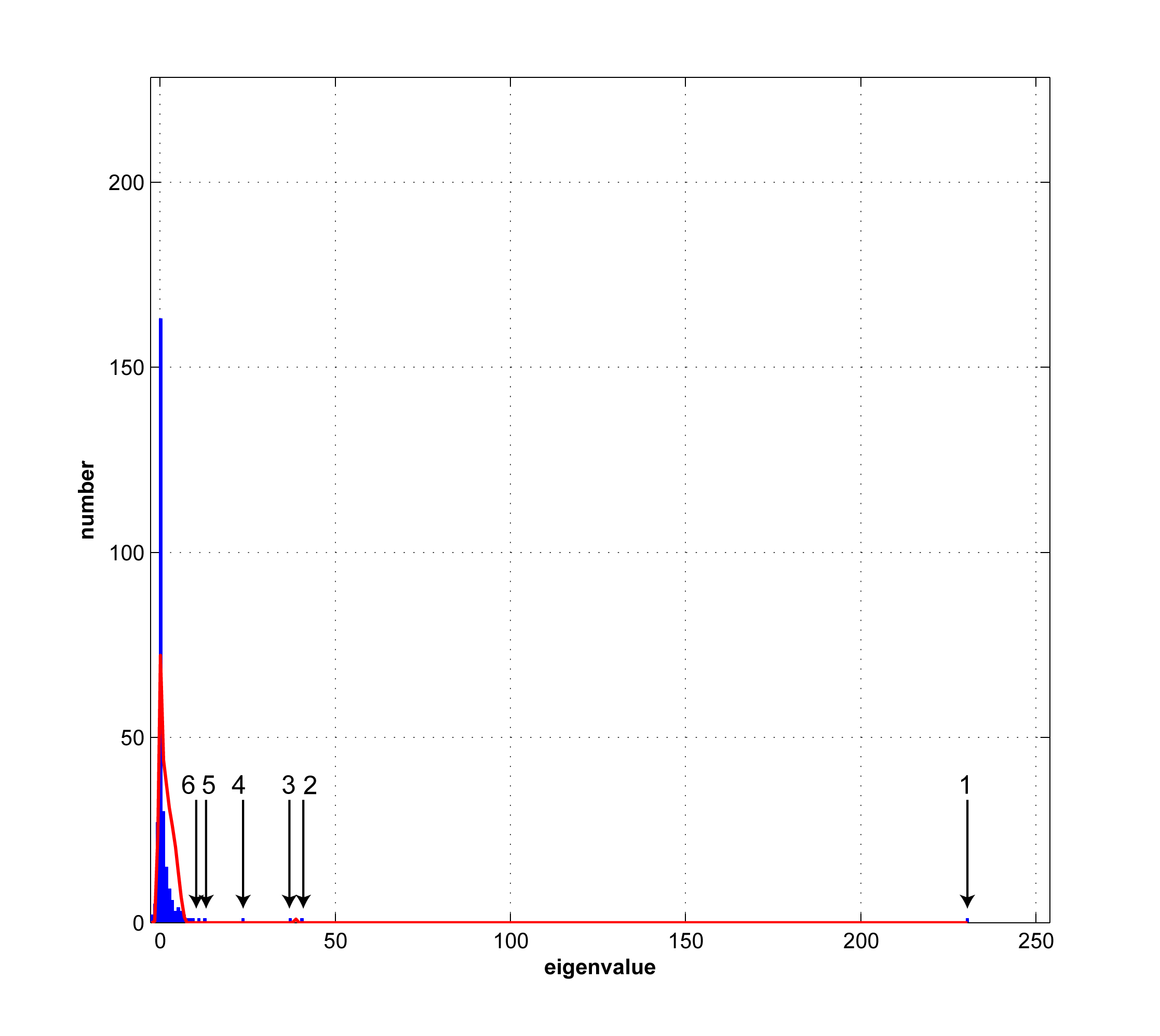

Supplement: Figure S1 — Histograms of the eigenvalues of the SCA positional correlation matrix. Histograms of the eigenvalues from decomposition of the positional correlation matrix for the BF2 MHC I heavy chain multiple sequence alignment in blue. The top six eigenmodes are indicated with arrows. Eigenvalues generated from decomposition of 100 randomized alignments are shown in red. (TIF) [file pone.0089657.s001.tif]

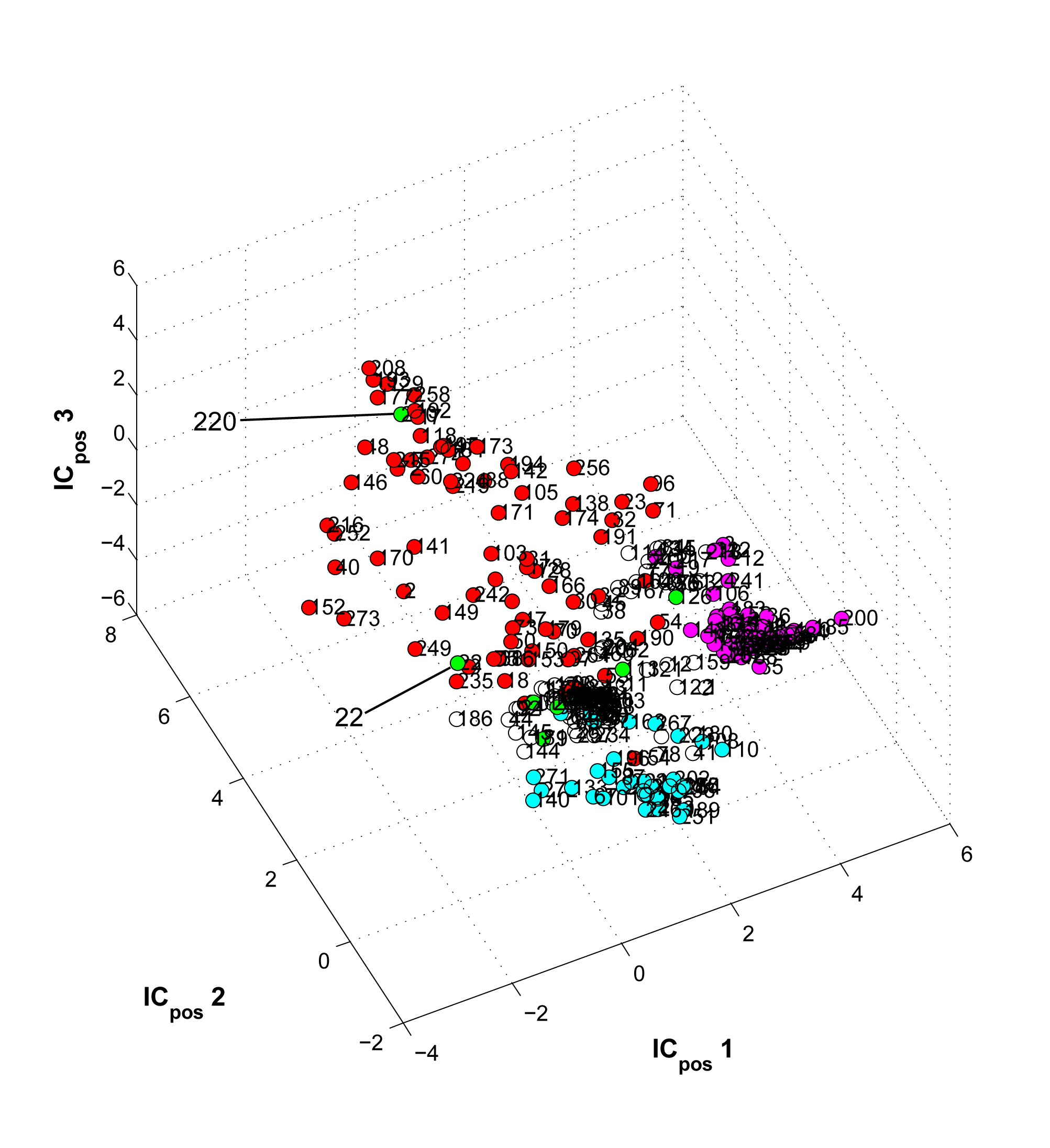

Supplement: Figure S2 — Identification of the protein sector by Independent Component Analysis. A plot of the top three independent components generated by transformation of the top six eigenmodes of the SCA matrix using Independent Component Analysis as previously described in [29], [41] to test for the existence of quasi-independent sectors. The identified protein sector is indicated along IC2 in red. The polymorphisms between BF2*15∶01 and BF2*19∶01 are shown in green. Two pusedo-sectors identified by the ICA are indicated in cyan and magenta. These putative sectors were discarded as they are not contiguous in the tertiary structure and most residues are close to zero. (TIF) [file pone.0089657.s002.tif]

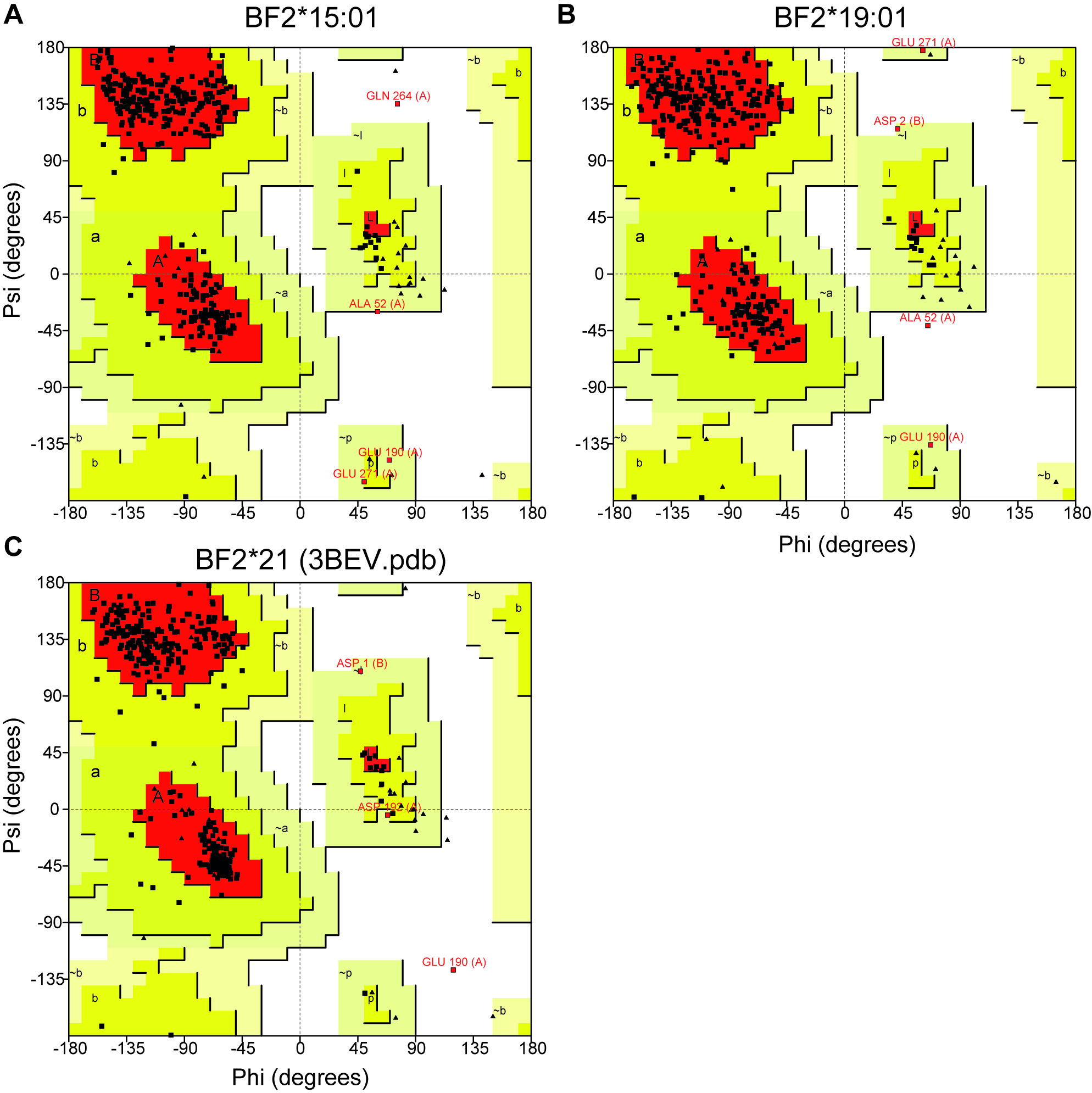

Supplement: Figure S3 — Ramachandran plots comparing the homology models of BF2*15∶01 and BF2*19∶01 to the template structure of BF2*21. Ramachandran plots indicating the conformational φ and ψ angles to assess the quality of the homology models of A) BF2*15∶01 and B) BF2*19∶01 in comparison to the crystallographic template structure of C) BF2*21 were generated using SWISS-MODEL incorporating PROCHECK [34]–[37]. (TIF) [file pone.0089657.s003.tif]

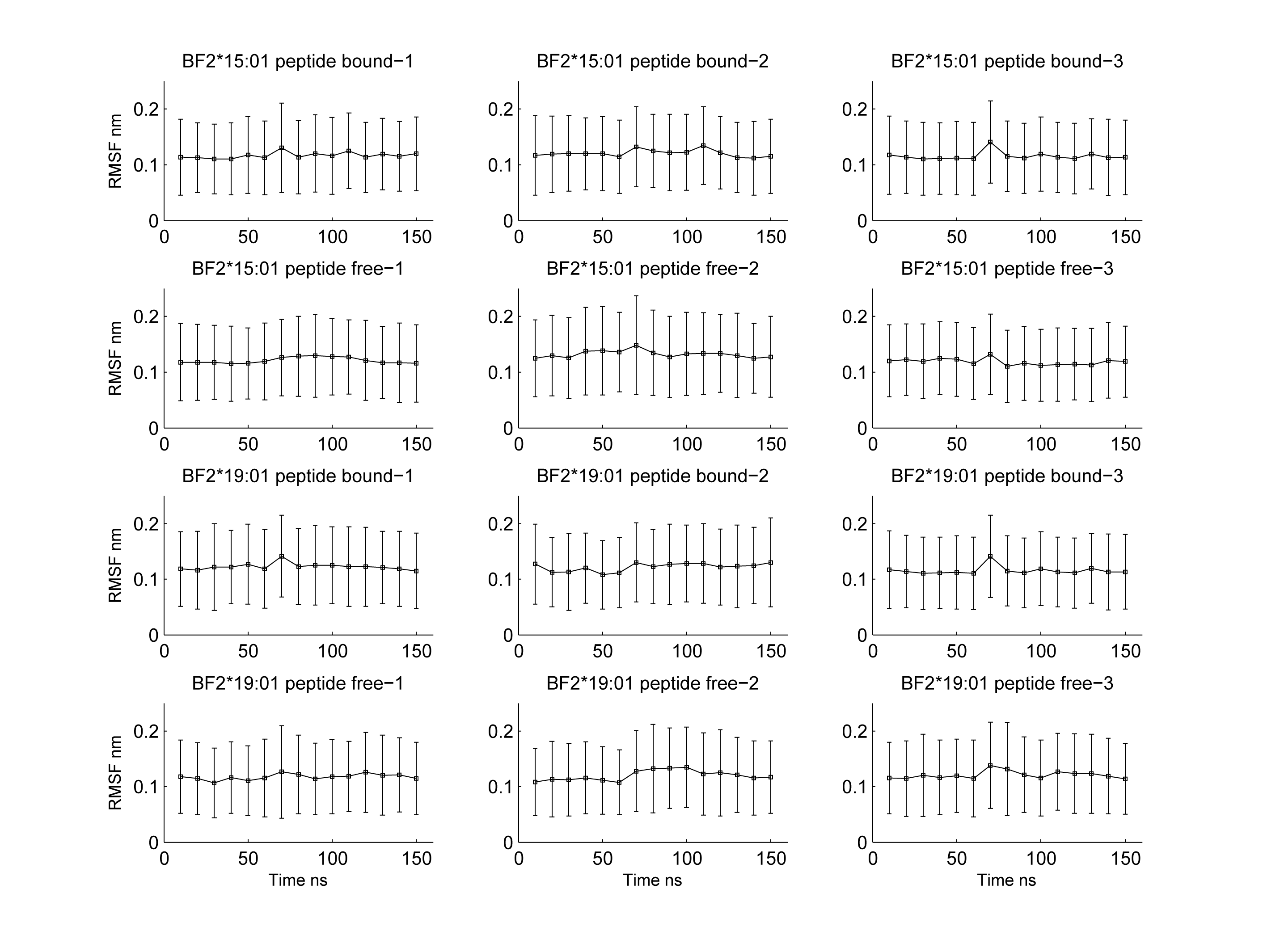

Supplement: Figure S4 — Time block assessment of the stability of the molecular dynamics simulations of BF2*15∶01 and BF2*19∶01. Each plot shows the Root Mean Square Fluctuation (RMSF) of the atoms from their average position during each 10 nanosecond time block of each molecular dynamics simulation trajectory as an indication of the overall stability of each simulation and between simulations. (TIF) [file pone.0089657.s004.tif]
