# Supplementary material for: Two Polymorphisms Facilitate Differences in Plasticity between Two Chicken Major Histocompatibility Complex Class I Proteins
Source: PLoS One. 2014 Feb 20;9(2):e89657. doi: 10.1371/journal.pone.0089657 (PMC3930747; doi:10.1371/journal.pone.0089657)
Supplement: Sector S1 — The protein sector identified by SCA for BF2 heavy chain using PDB residue numbering. (DOC) [file pone.0089657.s006.doc]

2+5+9+17+18+19+22+23+30+32+40+46+47+48+50+53+54+60+68+70+71+72+73+75+80+81+82+88+96+97+100+103+105+106+116+118+128+129+135+138+141+142+146+149+150+152+153+154+166+170+171+173+174+177+178+179+183+184+188+190+191+192+193+194+195+208+215+216+219+220+224+235+242+247+249+252+256+258+260+264+273+274+
