# Supplementary material for: Two Polymorphisms Facilitate Differences in Plasticity between Two Chicken Major Histocompatibility Complex Class I Proteins
Source: PLoS One. 2014 Feb 20;9(2):e89657. doi: 10.1371/journal.pone.0089657 (PMC3930747; doi:10.1371/journal.pone.0089657)

BF2*15:01
pepitde bound PC1

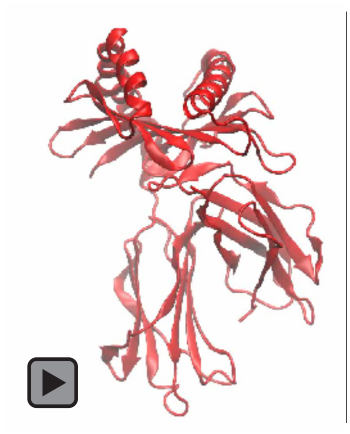

BF2*15:01
pepitde bound PC2

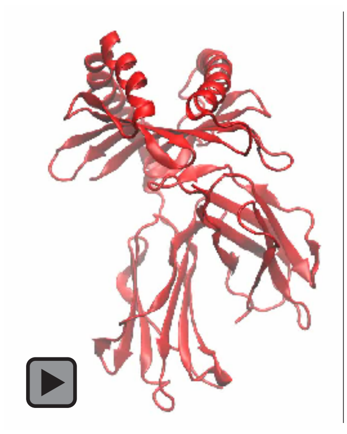

BF2*15:01
pepitde free PC1

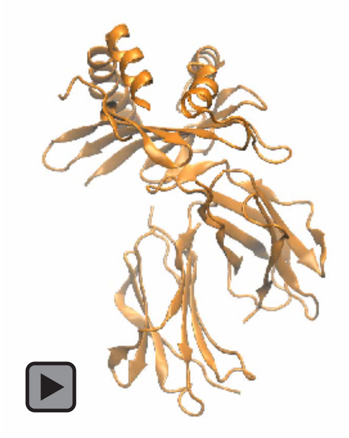

BF2*15:01
pepitde free PC2

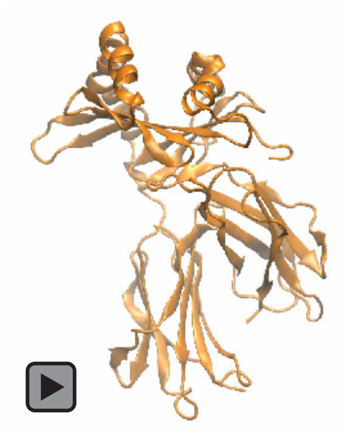

BF2*19:01
peptide bound PC1

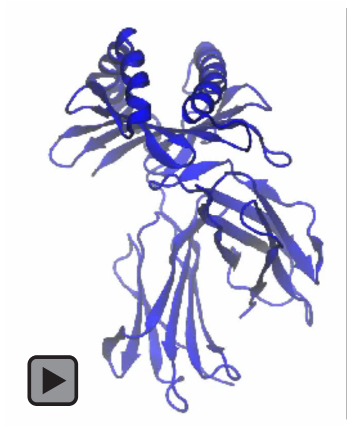

BF2*19:01
peptide bound PC2

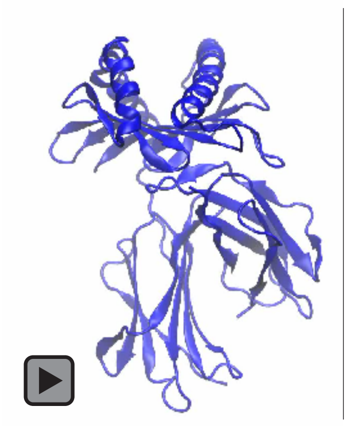

BF2*19:01
peptide free PC1

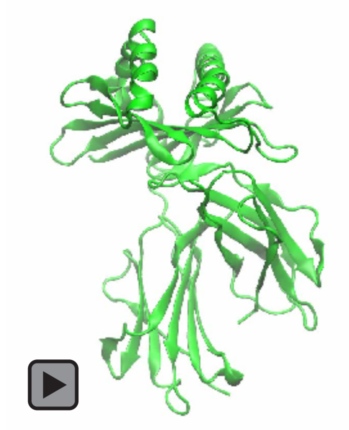

BF2*19:01
peptide free PC2

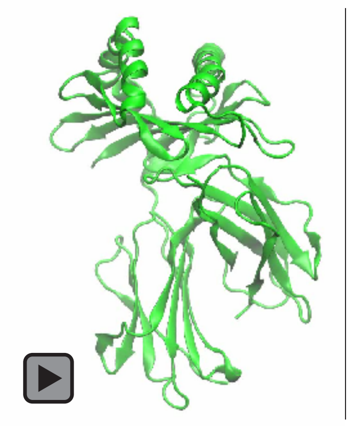

Supplement: Movies S1 — Animations of molecular dynamics simulations of BF2*15∶01 and BF2*19∶01 projected onto the first two principal components. The peptide is removed so that a common structure is used for the projections. The magnitude of the motions for BF2*15∶01 peptide free PC1 has created the appearance of a broken molecule. This is an artefact of the rendering. (PDF) [file pone.0089657.s007.pdf]
